# The Relationship between Circulating Kidney Injury Molecule-1 and Cardiovascular Morbidity and Mortality in Hemodialysis Patients

**DOI:** 10.3390/biomedicines12081903

**Published:** 2024-08-20

**Authors:** Alexandru Florin Sircuța, Iulia Dana Grosu, Adalbert Schiller, Ligia Petrica, Viviana Ivan, Oana Schiller, Madalina Bodea, Monica-Nicoleta Mircea, Ionuţ Goleț, Flaviu Bob

**Affiliations:** 1 Department of Internal Medicine II—Nephrology University Clinic, “Victor Babeș” University of Medicine and Pharmacy, Eftimie Murgu Sq. No. 2, 300041 Timișoara, Romania; alexandru.sircuta@umft.ro (A.F.S.); schiller.adalbert@umft.ro (A.S.); petrica.ligia@umft.ro (L.P.); maxim_mada@yahoo.com (M.B.); bob.flaviu@umft.ro (F.B.); 2 Centre for Molecular Research in Nephrology and Vascular Disease, Faculty of Medicine, “Victor Babes” University of Medicine and Pharmacy, Eftimie Murgu Sq. No. 2, 300041 Timișoara, Romania; 3 County Emergency Hospital, L. Rebreanu Street, Nr. 156, 300723 Timișoara, Romania; ivan.viviana@umft.ro; 4 Department of Internal Medicine II—Cardiology University Clinic, “Victor Babeș” University of Medicine and Pharmacy, Eftimie Murgu Sq. No. 2, 300041 Timișoara, Romania; 5 B Braun Avitum Dialysis Centre, 300417 Timișoara, Romania; oana.schiller@bbraun.com; 6 Institute of Cardiovascular Diseases, 300310 Timișoara, Romania; mircea.monica@yahoo.ro; 7 Department of Management, Faculty of Economics and Business Administration, University of the West, 300115 Timișoara, Romania; ionut.golet@e-uvt.ro

**Keywords:** plasma KIM-1, chronic kidney disease (CKD), hemodialysis (HD) patients, cardiovascular disease (CVD), left ventricular hypertrophy (LVH), CRP (C-reactive protein), IL-6 (interleukin-6), anemia

## Abstract

Background: The importance of identifying mortality biomarkers in chronic kidney disease (CKD), and especially in patients treated with hemodialysis (HD), has become evident. In addition to being a marker of tubulointerstitial injury, plasma kidney injury molecule-1 (KIM-1) has been mentioned in regard to HD patients as a risk marker for cardiovascular (CV) mortality and coronary artery calcification. The aim of this study was to assess the level of plasma KIM-1 as a marker of cardiovascular disease (CVD) and mortality in CKD5-HD patients (patients with CKD stage G5D treated with hemodialysis). Methods: We conducted a prospective case–control study that included 63 CKD5-HD patients (HD for 1–5 years) followed up for 48 months and a control group consisting of 52 non-dialysis patients diagnosed with CKD stages G1-G5 (ND-CKD). All patients had a CVD baseline assessment including medical history, echocardiography, and electrocardiography (ECG). Circulating plasma KIM-1 levels were determined with single-molecule counting immunoassay technology using an enzyme-linked immunosorbent assay. We obtained the following parameters: serum creatinine and urea; the inflammation markers CRP (C-reactive protein) and IL-6 (interleukin-6); and the anemia markers complete blood count, serum ferritin, and transferrin saturation (TSAT). Results: The mean plasma KIM-1 level was 403.8 ± 546.8 pg/mL, showing a statistically significant correlation with inflammation (CRP, R = 0.28, *p* = 0.02; IL-6, R = 0.36, *p* = 0.005) and with anemia (hematocrit, R = −0.5, *p* = −0.0316; hemoglobin (Hb), R = −0.5, *p* = 0.02). We found that patients with left ventricular hypertrophy (LVH) on echocardiography (59.7%) had significantly lower mean levels of plasma KIM-1 than patients from the control group (155.51 vs. 432.12 pg/mL; *p* = 0.026). Regarding the patients’ follow-up, we assessed all-cause mortality as an endpoint. After 24 months of follow-up, we found a mortality rate of 22.23%, while after 48 months, the mortality rate was 50.73%. A plasma KIM-1 level < 82.98 pg/mL was significantly associated with decreased survival in hemodialysis patients (*p* < 0.001). Conclusions: In patients treated with hemodialysis, low levels of plasma KIM-1 were associated with cardiovascular changes and an increased risk of mortality. Plasma KIM-1 levels were significantly higher in HD patients compared to ND-CKD patients.

## 1. Introduction

Chronic kidney disease (CKD) is a chronic and progressive disease that currently affects over 800 million people globally and has become one of the most important causes of death in the world [1]. Currently, CKD has become a major health issue, estimated to become the fifth cause of years lost due to disability by 2040 [2,3].

Despite recent medical advances, patients treated with hemodialysis present an increased cardiovascular (CV) risk, and cardiovascular disease (CVD) remains the main cause of death for these patients, with over 50% mortality in certain regions [4,5]. Therefore, it is of great importance to identify reliable biomarkers, both for renal injury and for predicting patients at risk of developing CV events.

Kidney injury molecule-1 (KIM-1) is a transmembrane glycoprotein that is considered to be increased during acute renal tubular injury since the molecule’s ectodomain is split and released in urine following proximal tubular injury. It is also identified as T-cell immunoglobulin mucin 1 and hepatitis A virus cellular receptor 1. Therefore, it is currently accepted as a urinary biomarker associated with an accelerated decline in renal function [6,7], but also with the risk of end-stage renal disease (ESRD) [8].

Since KIM-1 is released into the circulation after a renal injury, it seems reasonable to believe that assessing plasma levels of KIM-1 could also be beneficial. The free form of KIM-1 that can be identified in plasma, similar to the urinary excreted form, has a molecular mass of approximately 90 kDa (kilodalton) [9]. There are published data regarding the use of plasma KIM-1 as a biomarker in patients with acute kidney injury, but also with chronic kidney disease [10]. In the present study, we investigated the use of plasma KIM-1 as a prognostic marker in hemodialysis (HD) patients.

## 2. Materials and Methods

We performed a single-center prospective case–control study to assess plasma KIM-1 levels and inflammation markers in 63 patients undergoing hemodialysis who were followed up for 48 months and in a control group of 52 patients with non-dialysis-dependent CKD.

### 2.1. Patient Population

The group of patients undergoing hemodialysis consisted of 63 CKD G5D patients (patients with chronic kidney disease on hemodialysis), with all patients undergoing maintenance hemodialysis from a single hemodialysis center (Avitum B. Braun Centre in Timisoara, Romania). Out of the 138 patients from the center, we included patients who signed the informed consent form and had been treated with chronic hemodialysis for more than 1 year and less than 5 years (mean dialysis vintage 3.3 ± 1 years). The patients scheduled in the next 3 months for renal transplantation, for a change in renal replacement method, or a change in dialysis center, as well as patients with heart failure with reduced ejection fraction (HFrEF), were excluded from this study. Additionally, patients with a history of infection in the last 6 months were excluded.

The control group consisted of 52 age-matched patients previously diagnosed with CKD stages G1–G5, with an echocardiography performed in the past (those with HFrEF were excluded), and who were regularly followed up in the nephrology outpatient clinic.

All procedures were carried out in accordance with the ethical standards of the institutional research committee and with the Declaration of Helsinki, and were approved by the Ethics Committee of VICTOR BABES UNIVERSITY OF MEDICINE AND PHARMACY, TIMISOARA, ROMANIA (protocol code 33/30.06.2021). This study was authorized by the Dialysis Center’s Ethics Committee. Prior to any study procedure being conducted, eligible patients were asked to provide written informed consent.

### 2.2. Clinical and Biochemical Evaluation

The blood of fasting (at least 4 h) patients was drawn from the arteriovenous fistula or central venous catheter just before the mid-week HD session for the group of patients undergoing hemodialysis, and from a peripheral vein for the control group. The specimens were kept at 4 °C for 1 h and centrifuged at 1000× *g* for 10 min. The resultant serum was stored in aliquots at −80 °C until assayed. The frozen samples were thawed, and the measurements were performed immediately.

We obtained the following parameters: serum creatinine, urea; inflammation markers: CRP (C-reactive protein), IL-6 (interleukin 6); anemia markers: complete blood count, serum ferritin, transferrin saturation (TSAT); plasma KIM-1.

Circulating plasma KIM-1 levels were determined with single-molecule-counting immunoassay technology using an enzyme-linked immunosorbent assay (Elabscience, Houston, TX, USA: Human KIM-1 (Kidney Injury Molecule 1) ELISA Kit, Cat. no. E-EL-H6029). The quantitative analysis was determined spectrophotometrically, with the absorbance being read at 450 nm, using the Varioskan Lux microplate reader (Thermo Scientific™, Waltham, MA, USA). The sandwich ELISA concept was utilized with this ELISA kit. An antibody specific to Human KIM-1 was already applied onto the micro ELISA plate included in this kit. The ELISA microplate wells were filled with samples (or standards) of the appropriate antibody. Each microplate well was subsequently filled with a biotinylated detection antibody specific for Human KIM-1 and Avidin Horseradish Peroxidase (HRP) conjugate, and incubated. Loose parts were removed through washing. The substrate solution was added to every well. The wells that showed up blue contained the Avidin HRP conjugate, biotinylated detection antibody, and Human KIM-1. Once a stop solution was added, the enzyme–substrate reaction was finished and the color changed to yellow. The optical density (OD) was assessed spectrophotometrically at a wavelength of 450 + 2 nm. The OD value was proportional to the concentration of Human KIM-1.

For each patient, we recorded demographical data (including gender, age), medical history, dialysis vintage, CKD etiology, history of comorbidities such as diabetes mellitus, presence of coronary heart disease, presence of stroke, presence of hepatobiliary pathology, and the use of concomitant medication.

At the beginning of this study, during the second and third hours of the dialysis session, electrocardiography (ECG) and echocardiography (pulsed Doppler, continuous and two-dimensional M-mode) were performed for all patients by the same operator using the same equipment (to minimize differences). Regarding ECG, signs of myocardial ischemia (flat or down-sloping ST-segment depression of 1.0 mm or greater) were recorded. Echocardiography was performed according to the recommendations of the European Association for Cardiac Imaging (EACI). Left ventricular ejection fraction (LVEF) was determined using the Simpson method. Heart valve calcification and fibrosis, endocardial calcification, left ventricular end-diastolic diameter (LVTDD), left ventricular end-systolic diameter (LVTSD), and global longitudinal strain (GLS) were detected. Information on the ventricular septum (IVS), left ventricular mass (LVM), aortic atheroma, left atrium (LA), right ventricle (RV), and I/O was also reported.

At the end of the dialysis session, the weight of the patients was obtained, and this dry weight value was used to assess the BMI (body mass index).

All data obtained at the baseline visit were included in an intermediate analysis, representing a retrospective analysis of the subjects included in this study.

### 2.3. Follow-Up

For all hemodialysis patients, we used all-cause mortality at 48 months after inclusion as an end point.

### 2.4. Statistical Analysis

The data are presented as average ± standard deviation for numerical variables with Gaussian distribution, median and interquartile range for numerical variables with non-Gaussian distribution, and percentage from the sub-group total and number of individuals.

We used MedCalc software, version 12.5.0 (MedCalc, Mariakerke, Belgium), for statistical analysis. We used the Kolmogorov–Smirnov test to assess the distribution of numeric variables. For comparing qualitative variables, we used Pearson’s chi-squared test and the Spearman rank correlation test. For group comparisons of continuous variables with a normal distribution, we used analysis of variance (ANOVA), Scheffé’s test for all pairwise comparisons, and Levene’s test for equality of error variances. We used the Jamovi project (2022) (Version 2.3) software, retrieved from https://www.jamovi.org, accessed on 2 July 2024, in order to calculate a cut-off for continuous variables based on survival outcome. After the cut-off was determined, median survivals and 1-, 3-, and 5-year survivals were calculated. Thus, we used quartiles to arrange the data from smallest to largest, and the quantiles served as cut-off points between each group.

In order to complete the statistical computing and graphics, R Core Team’s (2021) R software (Version 4.1) was used. Log-rank, Gehan, Tarone–Ware, and Peto–Peto tests were used to compare the analysis of the differences.

## 3. Results

In this study, 63 patients with CKD stage G5D (treated with hemodialysis) and 52 non-dialysis CKD patients (ND-CKD) were enrolled. The characteristics of the studied patient groups are presented in Table 1 and Table 2.

### 3.1. Plasma KIM-1

The mean plasma KIM-1 levels were 403.8 ± 546.8 pg/mL in the studied group of HD patients. Comparing the HD group with the ND-CKD group, no statistically significant differences were detected in terms of age, gender, or inflammation status.

In the HD group, the plasma KIM-1 levels showed a statistically notable correlation with inflammation (mean CRP: R = 0.28, *p* = 0.02; IL6: R = 0.36, *p* = 0.005) and with anemia (hematocrit: R = −0.5, *p* = −0.0316; hemoglobin: R = −0.5, *p* = 0.02) (Table 3).

Regarding CKD etiology in HD patients, we found that patients with glomerular diseases (42 patients with diabetic kidney disease or glomerulonephritis) had higher, statistically non-significant, values of plasma KIM-1 compared to patients with non-glomerular diseases (21 patients) (528.08 ± 661.38 pg/mL vs. 209.69 ± 177.41 pg/mL, *p* = 0.06).

The level of plasma KIM-1 was not influenced by dialysis efficiency (expressed as kT/V: mean level 1.3 ± 0.3). No statistically significant differences were found regarding plasma KIM-1 levels in patients with kT/V > 1.3 compared to those that did not reach the target (359.4 ± 503.3 vs. 466.4 ± 613.3 pg/mL, *p* = 0.5).

**Figure 1:**
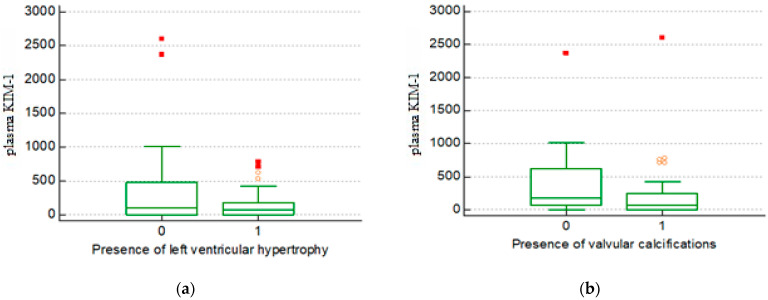
(**a**) Plasma KIM-1 levels in patients with and without left ventricular hypertrophy on echocardiography; (**b**) plasma KIM-1 levels in patients with and without valvular calcifications on echocardiography.

Left ventricular hypertrophy (LVH) was present in 37 out of 63 patients (58.7%), while 48 out of 63 patients (76.1%) had valvular calcifications on echocardiography. Using an ANOVA, we found that patients with LVH had considerably reduced mean levels of plasma KIM-1 (155.51 vs. 432.12 pg/mL; *p* = 0.026) (Figure 1a). Additionally, patients with valvular calcifications had reduced levels of serum KIM-1 (210.01 vs. 462.58 pg/mL, *p* = 0.04) (Figure 1b). There was no statistically significant similarity between plasma KIM-1 and ejection fraction, but patients with an ejection fraction below 40% were excluded from this study. We failed to identify any statistically significant relationship between the other CVD markers (coronary heart disease, cerebrovascular disease) and the level of plasma KIM-1 (Table 4).

### 3.2. Survival Analysis

**Figure 2:**
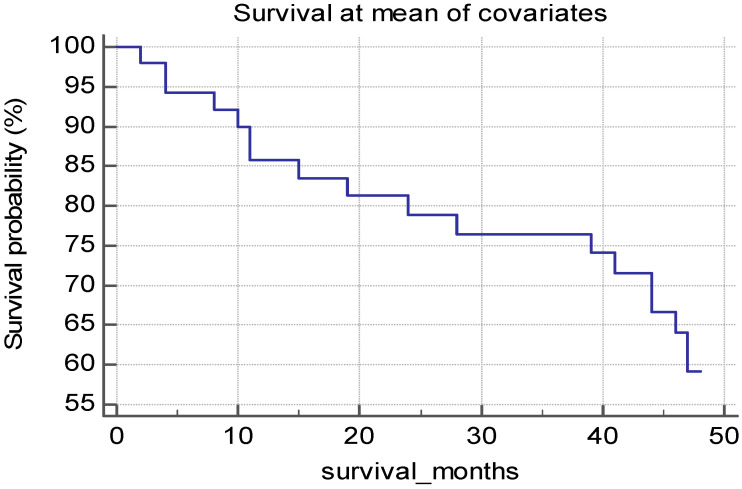
Survival probability of the HD patients.

Regarding the follow-up of the HD patients, we assessed all-cause mortality as an endpoint. After 24 months of follow-up, we found a mortality rate of 22.23%, while after 48 months, the mortality rate was 50.73% (Figure 2). The cause of death was cardiovascular in 8 patients (12.6%) and infectious in 11 patients (17.4%).

The mortality rate was calculated from the study inclusion criteria. The mortality after dialysis onset in this group of patients was 20.9% after 5 years of HD.

Using a Cox proportional hazards regression analysis of the predicting factors of mortality, we found that some cut-off values were associated with a significantly lower probability of survival. After the cut-off was determined, the median survival and 1-, 3-, and 5-year survivals were calculated.

**Figure 3:**
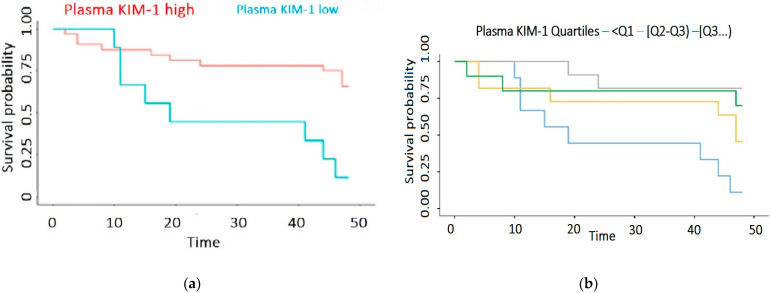
(**a**) Cox regression analysis of the influence of plasma KIM-1 on survival of HD patients; (**b**) Cox regression analysis of the quartiles of plasma KIM-1 levels.

Regarding the relationship between the plasma levels of KIM-1 and mortality, we found a significantly decreased survival rate in patients with low KIM-1, i.e., <81.98 pg/mL (*p* < 0.001), with a cut-off point for plasma KIM-1 levels of 81.98 pg/mL. When plasma KIM-1 was high, the 12-month survival was 87.5% [77–99.74%, 95% CI]; likewise, for high values of plasma KIM-1, the 36-month survival was 78.1% [65–93.85%, 95% CI]. When the plasma KIM-1 was low, the 12-month survival was 66.7% [42–100.00%, 95% CI] and the 36-month survival was 44.4% [21–92.27%, 95% CI] (Figure 3a,b).

The plasma KIM-1 values were split into four equal groups using the quartile values Q1–Q3. In Figure 3b, the survival probability function (Kaplan–Meier) is shown for each of the abovementioned groups.

In the first 12 months, the survival curve hit a peak, and then fluctuated widely, decreasing significantly after 12 months and reaching the lowest point after 24 months.

**Figure 4:**
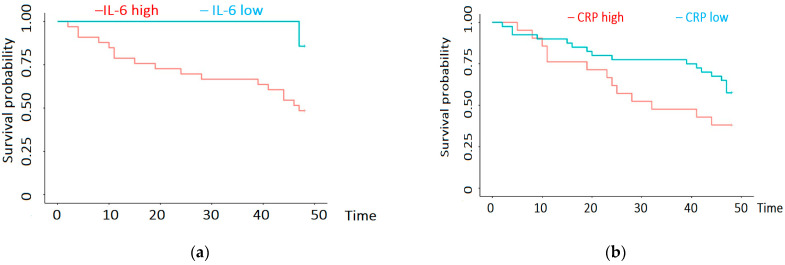
(**a**) Cox regression analysis of the influence of plasma IL-6 on survival of patients treated with hemodialysis; (**b**) Cox regression analysis of the influence of plasma CRP on survival of HD patients.

Regarding inflammation, we found significantly decreased survival in patients with IL-6 > 9.8 pg/mL. Thus, when IL-6 levels were high, the 12-month survival was 79% [66–94.0%, 95% CI] and the 36-month survival was 67% [52–84.9%, 95% CI]. For low levels of IL-6, the 12- and 36-month survival was 100% [100–100.0%, 95% CI] (Figure 4a). Regarding CRP, we found significantly decreased survival when CRP was over 1.22 mg/dL. When CRP was high, the 12-month survival was 76.2% [60.0–96.8%, 95% CI] and the 36-month survival was 47.6% [30.4–74.6%, 95% CI]. For low CRP, the 12-month survival was 90.0% [81.2–99.8%, 95% CI] and the 36-month survival was 77.5% [65.6–91.6%, 95% CI] (Figure 4b).

**Figure 5:**
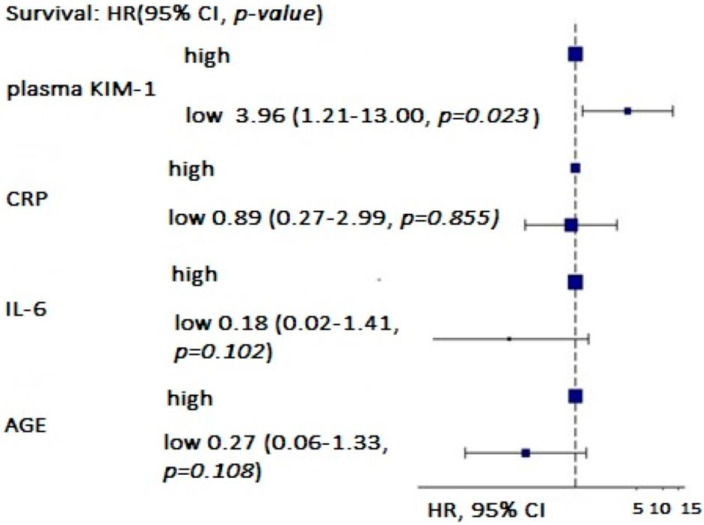
Multivariate analysis (hazard regression plot, 95% confidence intervals) of the influence of age, plasma KIM-1, CRP, and IL-6 on survival of HD patients.

In a multivariate survival analysis, low plasma KIM-1 remained a statistically significant predictor of mortality, after adjusting for inflammation (CRP and IL-6) and age (*p* = 0.023) (Figure 5).

The log-rank test was used to determine the optimal cut-off values for all significant variables with the objective of maximizing the difference in the survival probability. This grouping procedure led to the creation of two groups for each variable: above the cut-off value (high) and below the cut-off value (low). The abovementioned grouping procedure generated ordered variables with two levels, which were subsequently used in the Cox proportional hazards regression analysis as explanatory variables. The results of the analysis (hazard ratios, 95% confidence intervals, and observed significance level) are shown in Figure 5.

## 4. Discussion

The importance of identifying reliable biomarkers of mortality in CKD, especially in patients treated with hemodialysis, has become evident in recent years. Considering the cardiovascular disease (CVD) burden that these patients have, it would be ideal to identify novel serum biomarkers related to both the heart and the kidney.

KIM-1 is usually expressed by the nephron in acute tubular injury, being an early marker of acute kidney injury. The role of urinary KIM-1 as a marker of tubular injury is well known, as it is one of the most commonly studied urinary biomarkers. Due to the fact that, in some HD patients, the residual urine output is extremely decreased, the utility of urinary biomarkers in these patients is limited; therefore, the use of plasmatic markers would be more useful.

Plasma KIM-1 was linked to renal injury and declining GFR (glomerular filtration rate); thereby, a correlation between plasma KIM-1 and urinary KIM-1 was described [10]. The increase in the serum tubular marker KIM-1 is related to its leakage into the circulation due to an increased transepithelial or microvascular permeability or to the direct release of KIM-1 into the interstitium. This would mean that plasma KIM-1 is also a marker of tubulo-interstitial injury, similar to urinary KIM-1 [7,10,11,12,13,14].

The first potential application of plasma KIM-1 as a marker seems to be for the assessment of acute kidney injury (AKI). In a study performed by Sabbisetti et al., plasma KIM-1 levels increased during AKI compared to healthy controls or post-cardiac surgery patients without AKI [15]. A study performed in patients with ANCA-associated vasculitis showed that plasma KIM-1 levels correlated not only with the extent of tubulointerstitial damage in these patients, but also with the response to treatment. Although KIM-1 is expressed in hepatocytes as well, the fact that there was no liver injury in these patients indicates the sole tubular origin of the biomarker [15]. A small study in patients with IgA vasculitis (IgAV) showed that the plasma KIM-1 level was significantly higher in IgAV patients with nephritis [13]. Nevertheless, the plasma KIM-1 levels were not considerably different between IgAV patients without nephritis and controls [13].

It seems that, in acute settings, KIM-1, also known as TIM-1 (T-cell immunoglobulin mucin domain 1), has anti-inflammatory properties, participating in the phagocytosis of dead cells [16].

In our patients, there was a direct relationship between the level of plasma KIM-1 and markers of inflammation (CRP and IL-6). However, in our patients, it seems that plasma KIM-1 had an independent influence on survival even after adjusting to the studied inflammation markers (CRP and IL-6).

Even though there are data regarding the use of KIM-1 plasma levels in CKD and their relationship with disease incidence and outcomes, the studies are quite heterogeneous [17].

Studies have shown that plasma KIM-1 in diabetic patients has prognostic value for CKD progression, with higher plasma levels linked to renal decline that starts prematurely in type 1 and 2 diabetes [6,12,18]. Moreover, it has been shown that treatment with a sodium-glucose transporter protein 2 (SGLT2) inhibitor gives rise to a drop in the plasma level KIM-1 in CKD G3 (stage 3 of chronic kidney disease) patients [19]. Similar findings were found in children in the Chronic Kidney Disease in Children (CKID) Study, which showed that plasma KIM-1, TNFR-1 (plasma TNF receptor 1), and TNFR-2 (plasma TNF receptor 2) concentrations were independently associated with CKD progression [14].

In a study performed in two independent CKD cohorts—the Boston Kidney Biopsy Cohort (BKBC) and the Chronic Renal Insufficiency Cohort (CRIC)—it was shown that higher plasma KIM-1 levels were linked to severe acute tubular injury, tubulointerstitial inflammation, and mesangial expansion. Participants with diabetic nephropathy, glomerulopathies, and tubulointerstitial disease had significantly higher KIM-1 levels. Each doubling of plasma KIM-1 increased the risk of kidney failure. There was, however, no relationship with the mortality of the patients [20].

In our study, the patients with glomerular diseases (diabetic kidney disease and glomerulonephritis) had higher levels of plasma KIM-1 compared to other causes of CKD; however, the differences were not statistically significant. Overall, the plasma KIM-1 in our patients treated with hemodialysis was higher than in patients with different degrees of ND-CKD. Therefore, the question arises as to what leads to this difference and what the significance is of plasma KIM-1 in HD patients. Even in predialysis patients, it seems that there is not always a link between plasma KIM-1 and different tubular injury marker (NGAL, neutrophil gelatinase-associated lipocalin, and L-FABP, L-type Fatty Acid Binding Protein) concentrations. Moreover, plasma KIM-1 levels can be found in healthy volunteers and, consequently, in the absence of renal (tubular) injury, according to a prior study in patients with kidney disease [10]. The explanation for why the level of plasma KIM-1 does not always correlate with other markers of tubular injury, such as NGAL, is the fact that KIM-1 is produced by other tissues as well (testis, liver, spleen, and intestine) [21]. This explains the high levels of KIM-1 in the circulation even in the absence of tubular injury. It is plausible that the heart or the lungs may release KIM-1 because of the pathological alterations linked to heart failure [21].

KIM-1 is reported in the literature as acting as a cardio-renal marker as well. In heart failure (HF) patients, the levels of urinary KIM-1 are increased and correlated with the severity of the disease, and they could be associated with long-term outcomes. This relationship may indicate the presence of tubular injury in chronic (HF) patients. The direct relationship between urinary and plasma KIM-1 could lead to the idea that a similar level of significance as a marker could be attributed to plasma KIM-1. In order to prove this relationship, Emmens et al. performed a study in patients with chronic and acute HF. The results showed an association between plasma KIM-1 and decreased renal function, but not with all tubular markers of damage. There was also only a limited correlation with the clinical outcome of HF, showing that it has limited value as a CV marker [21]. Similarly, in the ASCEND-HF (Acute Study of Clinical Effectiveness of Nesiritide in Decompensated Heart Failure) trial performed in 874 patients with decompensated HF, circulating KIM-1 at baseline and during hospitalization was not associated with adverse clinical outcomes [11].

However, in a study performed on 90 HD patients, it was shown that there were elevated KIM-1 levels in the CKD5-HD patients with HF compared to those without HF [22]. The association with increased NT pro-BNP (N-terminal pro b-type natriuretic peptide) levels led to the idea that natriuretic peptides and KIM-1 may contribute to the pathogenesis of HF in CKD5-HD patients [22]. It is difficult to determine whether the elevation of KIM-1 in patients with HF is the result of volume and pressure overload or if it is a non-hemodynamic factor involved in the remodeling and dysfunction of the myocardium [22].

In a proteomic analysis of three cohorts of hemodialysis patients, plasma KIM-1, compared to 91 other proteins, was identified as a risk marker for CV mortality and coronary artery calcification in all three independent cohorts [23]. The authors of the study conclude that KIM-1 may originate from tissues other than the kidney, and could have a potential pathogenic role in premature vascular aging processes [23].

In our study, we assessed the relationship between plasma KIM-1 and CV features. No statistically significant relationship was found between the level of plasma KIM-1 and ejection fraction or the presence of coronary heart disease. Valvular calcifications were present in 77,4% of our patients, and their presence correlated with low levels of plasma KIM-1. It is known that valvular calcifications are very prevalent and predict mortality and morbidity, as they are one of the main CV complications in HD patients [24,25]. This indicates the fact that the presence of valvular calcification, regardless of position, was associated with higher mortality in our patients.

Another echocardiographic feature related to mortality is left ventricular hypertrophy (LVH), which was diagnosed in 59.7% of our patients and was also surprisingly correlated with low plasma KIM-1 levels. An evaluation of the clinical outcomes of HD patients with varying LVH geometric patterns and severity revealed that patients with severe concentric LVH were at the highest risk of CV mortality, followed by mild to moderate concentric LVH, severe eccentric LVH, and mild to moderate eccentric LVH; a similar trend was observed for all-cause mortality [26].

Overall, we found that the high plasma KIM-1 levels in our patients were associated with better 4-year survival, possibly explained by the indirect relationship with the mentioned CV complications (valvular calcifications and LVH). It is noteworthy that patients with HFrEF were excluded from this study.

This could also explain why the all-cause mortality in our patients after the onset of dialysis, and not after the study inclusion, was significantly lower compared to published data (20.9% vs. 58.2% at 5 years of hemodialysis) [27].

The present study had some limitations. First, the number of patients was small, and we did not include a control group of subjects with normal renal function. Another limitation is the fact that information regarding the residual renal function of patients is not available on a regular basis, and, therefore, it could not be included in the statistical analysis. Moreover, the fact that the ND-CKD group could not be assessed dynamically due to the high “lost-to-follow-up” rate in these patients is another important limitation of this study. Even though additional studies are required to clarify the exact role of KIM-1 in hemodialysis patients, nevertheless, this discovery suggests that low plasma KIM-1 levels are correlated with a higher mortality rate. This small study, by its nature, is hypothesis-generating and certainly makes way for larger prospective cohorts to explore the validity of these findings.

## 5. Conclusions

The novelty of our study is the use of plasma KIM-1 levels as markers to predict cardiovascular morbidity and mortality in patients treated with hemodialysis. The plasma KIM-1 levels were significantly higher in patients treated with hemodialysis compared to the control group (ND-CKD patients). Remarkably, low levels of plasma KIM-1 were linked to certain cardiovascular changes, and also to an increased risk of mortality. Further investigations are required to establish the accuracy of plasma KIM-1 as a biomarker for use in clinical practice with hemodialysis patients.

## Figures and Tables

**Table 1:** Characteristics of the studied patients.

Parameter	Patients Treated with HD (Mean Values ± Standard Deviation)
Age (years)	60.1 ± 11.8
Female:Male	21:42
Hemoglobin (g/dL)	10.9 ± 1.0
Hematocrit (%)	33.3 ± 4.3
Serum ferritin (ng/mL)	995.9 ± 358.0
TSAT (%)	30.6 ± 10.5
Serum creatinine (predialysis) mg/dL	8.4 ± 1.9
Serum urea (predialysis) mg/dL	123.1 ± 28.7
Plasma KIM-1 (pg/mL)	403.8 ± 546.8
IL-6 (pg/mL)	10.8 ± 0.9
CRP (mg/dL)	1.0 ± 0.9

Legend: HD, hemodialysis; TSAT, transferrin saturation; CRP, C-reactive protein; IL-6, interleukin 6. The results are expressed as mean values ± standard deviations.

**Table 2:** Characteristics of the studied patients and comparison between groups.

Parameter	HD Patients (Mean Values ± Standard Deviation)	Control Group (Mean Values ± Standard Deviation)	Comparison between Groups (*p*-Value)
Age (years)	60.1 ± 11.8	59.03 ± 14	0.65
Female: Male	21:42	32:29	N.A.
Plasma KIM-1 (pg/mL)	403.8 ± 546.8	217.48 ± 267.10	0.02
IL-6 (pg/mL)	10.8 ± 0.9	9.5 ± 7.6	0.18
CRP (mg/dL)	1.0 ± 0.9	1.28 ± 2.97	0.47

Legend: HD, hemodialysis; CRP, C-reactive protein; IL-6, interleukin 6. The results are expressed as mean values ± standard deviations.

**Table 3:** Correlation between plasma KIM-1 level and the different parameters in HD patients.

Parameter	Correlation Coefficient
Age	NS
Hemoglobin	R = −0.5; *p* = 0.01
Hematocrit	R = −0.5; *p* = 0.01
Serum ferritin	NS
TSAT	NS
Serum creatinine (predialysis)	NS
Serum urea (predialysis)	NS
CRP	R = 0.28; *p* = 0.02
IL-6	R = 0.35; *p* = 0.005
kT/V	NS
Ejection fraction	NS

Legend: TSAT, transferrin saturation; CRP, C-reactive protein; IL-6, interleukin 6; kT/V, measurement of dialysis efficacy.

**Table 4:** Influence of different parameters on the level of plasma KIM1.

Parameter	Number of Patients (Out of 63)	Plasma KIM-1: Mean Value ± Standard Deviation (Comparison between Patients with and without the Parameter)	Correlation Coefficient
Valvular calcifications LVH	48	210.0 ± 414.1 vs. 462.5 ± 648.8	0.04
	37	155.5 ± 214.0 vs. 432.1 ± 690.2	0.02
Signs of ischemia on ECG Coronary heart disease	25	268.09 ± 515.06 vs. 266.34 ± 467.17	0.4
	32	291.33 ± 516.62 vs. 244.286 ± 456.13	0.7
Cerebrovascular disease Diabetes mellitus	8	109.9 ± 244.47 vs. 290.33 ± 506.17	0.97
	27	219.16 ± 436.13 vs. 329.11 ± 539.68	0.37
Male gender Glomerular disease	41	305.29 ± 562.06 vs. 192.37 ± 265.54	0.38
	42	528.08 ± 661.38 vs. 209.69 ± 177.41	0.06

ANOVA used for determination. Legend: ECG, electrocardiogram; LVH, left ventricular hypertrophy.

## Data Availability

The data presented in this study are available on request from the corresponding author.
